# Evaluation of Intralymphatic Immunotherapy in Allergic Rhinitis Patients: A Systematic Review and Meta-analysis

**DOI:** 10.1155/2023/9377518

**Published:** 2023-05-08

**Authors:** Sijie Jiang, Shaobing Xie, Qingping Tang, Hua Zhang, Zhihai Xie, Junyi Zhang, Weihong Jiang

**Affiliations:** ^1^Department of Otolaryngology Head and Neck Surgery, Xiangya Hospital of Central South University, Changsha, China; ^2^Hunan Province Key Laboratory of Otolaryngology Critical Diseases, Xiangya Hospital of Central South University, Changsha, China; ^3^National Clinical Research Center for Geriatric Disorders, Xiangya Hospital of Central South University, Changsha, China; ^4^Department of Rehabilitation, Brain Hospital of Hunan Province, Hunan University of Chinese Medicine, Changsha, China

## Abstract

**Background:**

Intralymphatic immunotherapy (ILIT) is short-course administration of allergen-specific immunotherapy (AIT). This study is aimed at assessing the clinical efficacy and safety of ILIT in patients with allergic rhinitis (AR).

**Methods:**

MEDLINE, PUBMED, and Cochrane Library were used to conduct electronic searches for clinical trials comparing ILIT and placebo in patients with AR. The final search took place on August 24, 2022. Cochrane Handbook for Systematic Reviews of Interventions was used to assess the risk of bias in the included studies. The outcomes included combined symptom and medication scores (CSMS), visual analog scale (VAS), allergic rhinoconjunctivitis quality of life (RQLQ), Skin-prick test (SPT), and adverse events (AEs). Data were synthesized as mean difference (MD)/standard mean difference (SMD) or risk difference (RD) and 95% confidence interval (CI).

**Results:**

Thirteen studies (454 participants) were included in this study. The ILIT group had better clinical improvement on the CSMS (random effects model, SMD-0.85, 95% CI [-1.58, -0.11], *P* = 0.02) and RQLQ (fixed-effects model, MD-0.42, 95% CI [0.69, 0.15], *P* = 0.003) than the placebo group. The booster injection was beneficial for CSMS (*P* < 0.0001), and the 4-week injection interval was superior to the 2-week injection period for improving VAS (*P* < 0.0001). Local swelling or erythema was the main AE following injection (random effects model, RD 0.16, 95% CI [0.05, 0.27], *P* = 0.005). *Discussion*. For individuals with AR, ILIT is safe and effective. ILIT alleviates clinical symptoms and reduces pharmaceutical consumption without causing severe AEs. However, the validity of this study is compromised by the substantial heterogeneity and risk of bias in the included researches. *Registration*CRD42022355329.

## 1. Introduction

Allergen-specific immunotherapy (AIT) is the only therapy currently available for changing the ordinary course of IgE-mediated allergic diseases [1, 2]. AIT offers the prospect of reducing allergic symptoms and improving quality of life by administering allergens to individuals who do not respond well to pharmaceutical treatments [3]. Traditionally, allergens were delivered subcutaneously (subcutaneous immunotherapy [SCIT]) or sublingually (sublingual immunotherapy (SLIT)) for at least three years to induce immunological tolerance and confer therapeutic advantages [3–5]. However, the lengthy treatment duration suggested increased expense and noncompliance with therapy [6, 7]. It is known that only secondary lymphatic organs, such as lymph nodes, can initiate immunological responses [8, 9]. Consequently, intralymphatic immunotherapy (ILIT) administers allergen extract directly to lymph nodes to induce rapid and effective immunological tolerance. Three injections are provided at 12-week intervals, resulting in fewer injections and a shorter treatment duration [10]. Therefore, ILIT is an alternate strategy to AIT that improves safety and efficacy for individuals with poor AIT adherence.

Previous clinical trials have demonstrated the efficacy of ILIT in treating perennial and seasonal allergen-related disorders [11, 12]. It has been suggested that applying ILIT benefited patients with asthma [13], atopic dermatitis [14], and allergic rhinitis (AR) [2]. However, the conclusions of the current AR study were inconclusive. Contrary to the findings of other investigations, Park et al. concluded that ILIT displayed indistinct therapeutic effects and moderate-to-severe systemic responses in AR [15]. Hitherto, prior meta-analysis proceeded with limited studies and participants, resulting in contradictory findings and relatively insufficient investigation of the safety and efficacy of ILIT with varying allergen dosages and follow-up periods [16, 17]. In light of this, the purpose of this systematic review is to evaluate the safety and efficacy of ILIT in patients with AR and the dose-time effect of ILIT based on current research.

## 2. Materials and Methods

This protocol for a systematic review and meta-analysis was submitted to PROSPERO (registration number: CRD42022355329). This research followed the Preferred Reporting Items for Systematic Reviews and Meta-Analyses (PRISMA) guidelines [18].

### 2.1. Eligibility Criteria

The clinical trials which conducted the comparison between ILIT and control groups were included. The inclusion criteria were as follows. (1) The diagnosis of AR was guided by the ARIA recommendations [3]. (2) ILIT with any allergen, dosage, preparation solution, treatment duration, and follow-up period were accepted. (3) The control group was administered the equivalent ILIT solvent. (4) Studies should report at least one of the following primary outcomes: combined symptom and medication scores (CSMS), visual analog scale (VAS), allergic rhinoconjunctivitis quality of life (RQLQ), skin prick test (SPT), and adverse events (AEs). Conference abstracts, secondary research projects, and animal experiments were excluded.

### 2.2. Search Strategy

Electronic searches were performed with MEDLINE, PUBMED, and Cochrane library. The search terms were “rhinitis, allergic,” “allergic rhinitis,” “hay fever,” “rhinoconjunctivitis,” “nasal allergy,” “lymph nodes,” “lymph^∗^,” “intralymph^∗^,” “intralymphatic immunotherapy,” and “injection, intralymphatic”. The last search was performed on August 24, 2022.

### 2.3. Study Selection and Data Extraction

Duplicate records were removed. Two reviewers (SJ and SX) independently screened the titles and the abstracts. The full-text versions of relevant studies were subsequently screened based on predetermined eligibility criteria for the final decision. Data extraction and collection were performed by two reviewers (QT and HZ) independently. All disagreements over the selection and extraction processes were resolved by thorough group discussion (ZX and HZ). The following data were extracted: first author name, year and country of publication, the number of participants, age, gender, allergen, dosage, treatment interval, booster dose, follow-up period, and outcome measurements. The corresponding author of the study with incomplete or ambiguous information was contacted for more data.

### 2.4. Data Items

Primary outcomes included CSMS, VAS, RQLQ, SPT, and AEs. The efficacy outcomes of seasonal allergic AR were evaluated in the pollen-peak season. AEs were recorded as total events number and injection number, categorized as local urticarial reaction, local swelling or erythema, abdominal pain or nausea, fatigue, eye or nasal symptoms, pulmonary symptoms, and headache.

### 2.5. Risk of Bias Assessment

The risk of bias in the included studies was evaluated in accordance with the Cochrane Handbook for Systematic Reviews of Interventions. Six items were considered, including random sequence generation (selection bias), allocation concealment (selection bias), blinding of participants and personnel (performance bias), blinding of outcome assessment (detection bias), incomplete outcome data (attrition bias), and selective reporting (reporting bias).

### 2.6. Synthesis Methods and Meta-analysis

The effect size of continuous data was presented as mean difference (MD) or standard mean difference (SMD) with a 95% confidence interval (CI). Dichotomous data were synthesized as risk difference (RD) with 95% CI. If data were supplied as median, range, 95% CI, and standard error, mean and standard deviation would be estimated with available information [19, 20]. Meta-analysis was performed with Review Manager, version 5.4 [21]. The *I*^2^ statistic was used to determine the heterogeneity of the analysis. When *I*^2^ was <40%, the heterogeneity was deemed “low”; and 40%-60% of *I*^2^ and >60% if *I*^2^ were deemed “moderate” and “substantial,” respectively [22]. When heterogeneity was low, a fixed-effects model was applied; otherwise, a random-effects model was employed, and subgroup analyses would be used to investigate the heterogeneity and enhance the robustness of the study.

## 3. Results

### 3.1. Study Selection

**Figure 1:**
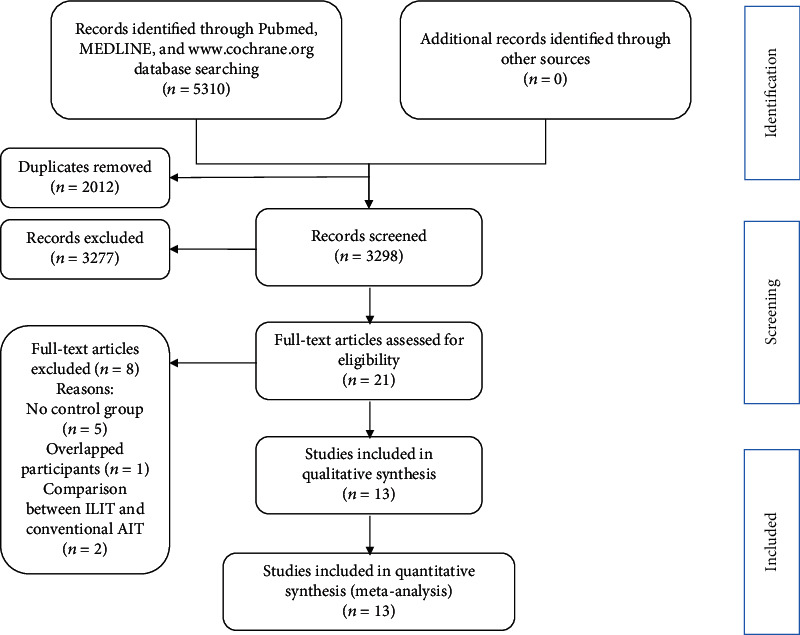
Flowchart of the study selection process.

A flowchart of the study selection process is profiled in Figure 1. Through database filtering and manual search, 5310 studies were obtained in total. After removing duplicates, 3298 records remained. Full texts of the 21 studies were retrieved and assessed, and eight were removed. In this section, eight studies were removed for the following reasons. Overlapping subjects were obtained in one study [23]. Five studies did not establish a control group [11, 12, 24–26]. Two studies compared ILIT with conventional AIT [27, 28]. Thirteen studies were included in the qualitative synthesis. There was another overlapping population in two studies that reported different outcomes [29, 30]. We incorporated and evaluated them into the final evaluation with respective clinical outcomes. Eleven studies [13, 15, 29–37] were included in the efficacy analysis, and ten [15, 29, 31, 32, 34–39] were included in the safety analysis.

### 3.2. Study Characteristics and Participants

**Figure 2:**
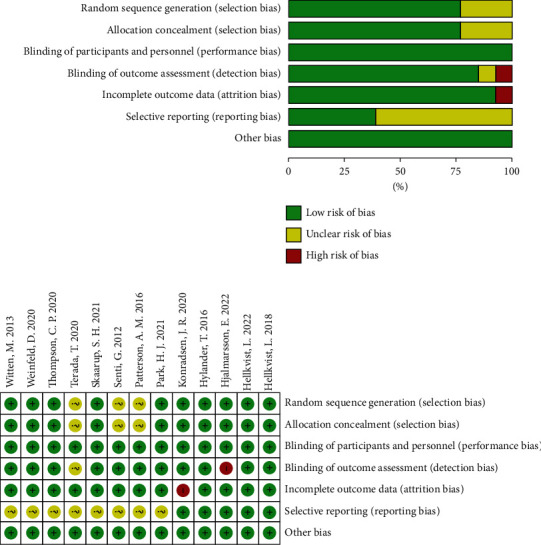
Risk of bias. (a) Risk-of-bias graph and (b) risk-of-bias summary.

Characteristics of the included studies are illustrated in Table 1. A total of 454 participants were included in this meta-analysis. The participants were allergic to seasonal (11 trials) and perennial allergens (2 trials). The intervention to seasonal AR was carried out before the onset of the pollen season.  Figure 2 depicts the estimations of the risk of bias in the included studies. There were unclear risks of bias in random sequence generation (23%), allocation concealment (23%), blinding of outcome assessment (8%), and selective reporting (62%). There were high risks of bias in the blinding of outcome assessment (8%) and incomplete outcome data (8%).

### 3.3. Outcomes

#### 3.3.1. Combined Symptom and Medication Scores (CSMS)

**Figure 3:**
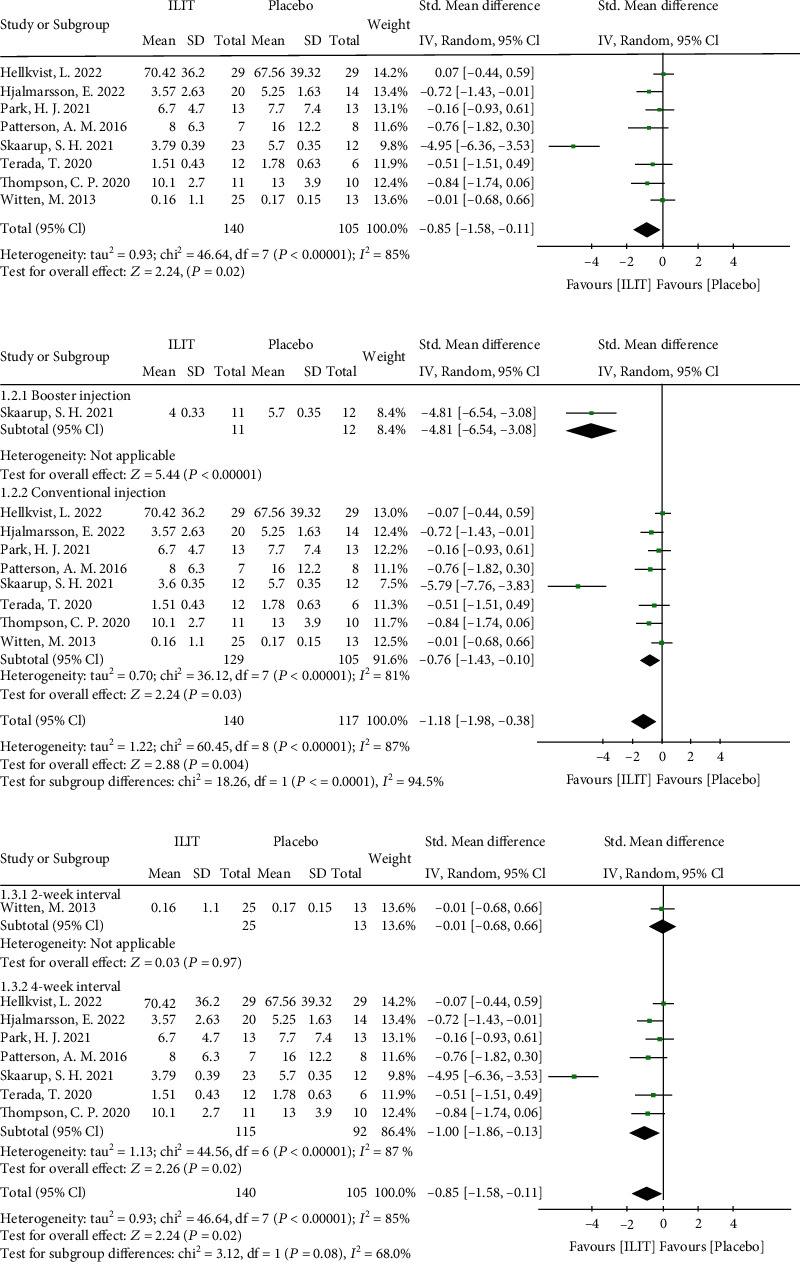
Combined symptom and medication scores. (a) Overall meta-analysis of CSMS; (b) subgroup analysis by booster injection; and (c) subgroup analysis by injection interval. ILIT: intralymphatic immunotherapy; CI: confidence interval; df: degrees of freedom; Std. mean difference: standardized mean difference.

Eight RCTs evaluated CSMS in AR patients between ILIT and placebo. The total number of patients for this outcome was 245. The effect size of the meta-analysis on the CMSM favored ILIT (random effects model, SMD -0.85, 95% CI [-1.58, -0.11], *P* = 0.02). The heterogeneity of this model was substantial (*I*^2^ = 85%) (Figure 3(a)). The forest plot of subgroup analysis by booster injection indicated the booster injection subgroup (random effects model, SMD -4.81, 95% CI [-6.54, -3.08], *P* < 0.0001) possessing significant improvement (*P* < 0.0001) in comparison to the conventional treatment group (random effects model, SMD -0.76, 95% CI [-1.43, -0.10], *P* = 0.03, *I*^2^ = 87%) (Figure 3(b)). Subgroup analysis by injection interval favored the 4-week interval group (random effects model, SMD -1.00, 95% CI [-1.86, -0.13], *P* = 0.02, *I*^2^ = 87%) rather than the 2-week interval group (random effects model, SMD -0.01, 95% CI [-0.68, 0.66], *P* = 0.97) (Figure 3(c)). Subgroup analysis by different dosages revealed no difference between the 3000 SQU group and the larger dosage group (*P* = 0.80) (Figure S1). To reduce heterogeneity, we excluded studies one by one and found that the heterogeneity was primarily from Skaarup et al. When we excluded this study, the *I*^2^ was reduced from 81% to 8% in the conventional treatment subgroup and 87% to 12% in the 4-week interval group (Figure S2-S3). The heterogeneity may be due to using different assessment methodologies before and after 2016 in Skaarup et al. The funnel plot is illustrated in Figure S4.

#### 3.3.2. Visual Analog Scale (VAS)

**Figure 4:**
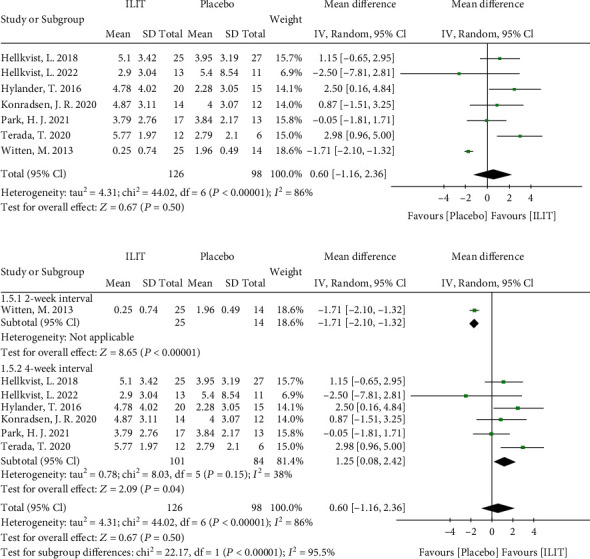
Visual analog scale. (a) Overall meta-analysis of VAS; (b) subgroup analysis by injection interval. ILIT: intralymphatic immunotherapy; CI: confidence interval; df: degrees of freedom.

Seven RCTs assessed the improvement of VAS of nasal symptoms, and 224 participants were included in this meta-analysis. The improvement of VAS between ILIT and placebo was insignificant (random effects model, MD 0.60, 95% CI [-1.16, 2.36], *P* = 0.50). The heterogeneity of this model was substantial (*I*^2^ = 86%) (Figure 4(a)). By a step-by-step exclusion procedure, we determined that the heterogeneity mainly originated from one study with a 2-week injection interval. Therefore, a subgroup analysis was undertaken by injection interval. Results showed that the improvement of VAS in the 4-week injection interval group (Random effects model, MD 1.25, 95% CI [0.08, 2.42], *P* = 0.04, *I*^2^ = 38%) was better (*P* < 0.0001) than the 2-week injection group (Random effects model, MD -1.71, 95% CI [-2.10, -1.32], *P* < 0.0001) (Figure 4(b)). Subgroup analysis by different dosages (*P* = 0.36) (Figure S5) and booster injection (*P* = 0.60) (Figure S6) showed no difference. The funnel plot is illustrated in Figure S7.

#### 3.3.3. Allergic Rhinoconjunctivitis Quality of Life (RQLQ)

**Figure 5:**
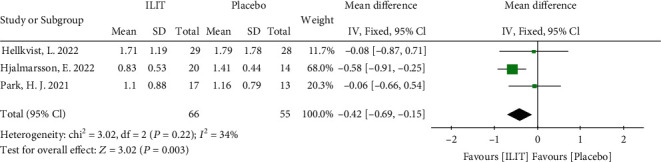
Overall meta-analysis of allergic rhinoconjunctivitis quality of life. ILIT: intralymphatic immunotherapy; CI: confidence interval; df: degrees of freedom.

Three RCTs assessed the RQLQ as outcomes. A total of 121 subjects were investigated. The meta-analysis supported that the RQLQ improvement in the ILIT group was superior to the placebo group (fixed-effects model, MD -0.42, 95% CI [-0.69, 0.15], *P* = 0.003). The heterogeneity of this model was low (*I*^2^ = 34%) (Figure 5). The funnel plot is demonstrated in Figure S8.

#### 3.3.4. Skin-Prick Test (SPT)

**Figure 6:**
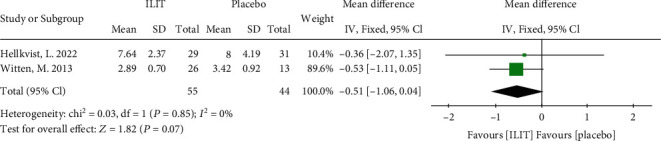
Overall meta-analysis of the skin-prick test. ILIT: intralymphatic immunotherapy; CI: confidence interval; df: degrees of freedom.

Two RCTs with 99 participants assessed the SPT as an outcome. The effect size of SPT between ILIT and placebo groups exhibited no statistical significance (random effects model, MD -0.51, 95% CI [-1.06, 0.04], *P* = 0.07). The heterogeneity of this model was low (*I*^2^ = 0%) (Figure 6(a)). The funnel plot is demonstrated in Figure S9.

#### 3.3.5. Adverse Events (AE)

**Figure 7:**
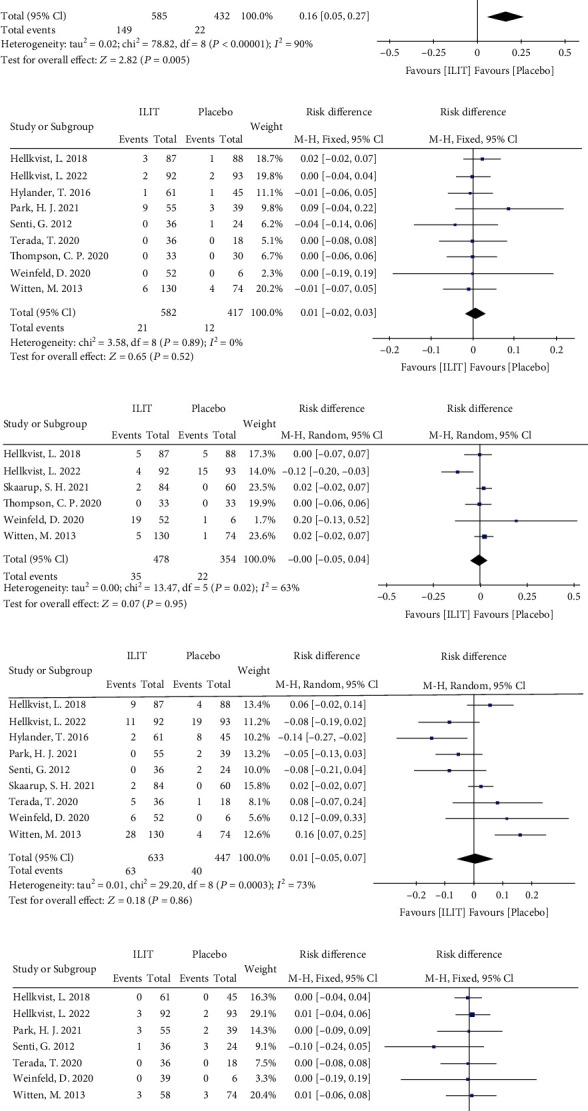
Adverse events after injection. (a) Local urticarial reaction, (b) local swelling or erythema, (c) abdominal pain or nausea, (d) fatigue, (e) eye or nasal symptoms, (f) headache, and (g) pulmonary symptoms. ILIT: intralymphatic immunotherapy; CI: confidence interval; df: degrees of freedom.

Ten trials with 1123 injection records reporting adverse events were included in the evaluation of the safety of ILIT. The meta-analysis favored ILIT in local swelling or erythema after injection (Random effects model, RD 0.16, 95% CI [0.05, 0.27], *P* = 0.005) with substantial heterogeneity (*I*^2^ = 90%). In addition, there were no significant differences between ILIT and placebo for local urticarial reaction, abdominal pain or nausea, fatigue, eye or nasal symptoms, headache, and pulmonary symptoms (*P* > 0.05) (Figures 7(a)–7(g)).

## 4. Discussion

This systematic review and meta-analysis included 13 trials with 454 participants and revealed that ILIT had positive impacts on CSMS and RQLQ improvements in AR patients. The subgroup analysis showed that the booster injection contributed to the enhancement of VAS. In terms of CSMS and VAS improvement, the 4-week injection period was superior to the 2-week injection interval.

Current guidelines recommend AIT as a curative treatment for allergic diseases, particularly for patients with a poor response to pharmacotherapies [40]. However, conventional AIT involves multifarious allergen administration protocols and requires good patient compliance. It came up with ILIT as an alternative option with more efficient schemes with limited evidence. Currently, available researches on the comparison between the conventional AIT and ILIT were insufficient to conduct a meta-analysis yet. However, current clinical trials have demonstrated that SLIT provides equivalent or superior therapeutic benefits to SCIT [28, 41]. Previous meta-analyses [42–44] have shown that relative to the placebo, the symptom score, and medication score both benefitted more from SCIT and SLIT. Meanwhile, this meta-analysis of ILIT strengthened the positive effects on the clinical outcomes, such as CSMS and RQLQ, by incorporating more high-quality RCTs compared with existing meta-analysis [16, 17].

The treatment period of ILIT treatment protocol is relatively short, and discussion has arisen about the potential effects of an extra preseasonal booster injection in the next year. Several trials showed that a preseasonal booster injection significantly alleviated symptoms of pollen-induced AR compared with both placebo and 3-injection ILIT [13, 39]. Consistent with prior researches, our subgroup analysis of CSMS and VAS by booster dosage revealed a significant advantage for the booster dosage group [9, 45]. These results suggested that a booster dose of ILIT could ameliorate the symptoms of seasonal AR and reduce medication consumption by boosting allergen affinity and developing immunological tolerance. Besides, one trial with a 2-week injection interval exhibited heterogeneity independent from other studies. The investigation of the immune response and immunological tolerance formation period has attracted considerable interest. In our subgroup analysis, the 4-week injection interval groups showed substantial improvements in CSMS and VAS compared to the 2-week injection interval groups. It was tempting to believe that a 4-week injection interval would be more suitable for producing suppressive immune cells and developing immunological tolerance. Based on these findings and previously published studies, a 4-week injection interval was recommended, and a pre-seasonal booster injection might contribute to increasing the effectiveness of ILIT.

According to prior research, the efficacy of AIT presented a dose response, while ILIT had an advantage in reducing the dosage of allergen extract [46, 47]. Concomitantly, the injection dosage may impact the therapeutic effectiveness of ILIT. Curiously, our subgroup analysis of CSMS and VAS by injection dosage revealed no significant difference across dosage-specific groups. However, considering the substantial heterogeneity in subgroup analysis, the results might not be clinically significant. We considered that in addition to the total dose, the injection interval and the single injection dose were crucial elements influencing the pharmaceutical efficacy. Conducting the injection at a 2-week interval was insufficient to induce immune tolerance, even with a larger total dosage, as described in the previous subgroup analysis. On the other side, there was considerable diversity in the administration protocols of the larger dosage group, which comprised various single dosages. All these factors might influence the effectiveness of ILIT. Hitherto, the research evaluating the dose-time effect of ILIT is still insufficient and encouraged.

AE was one of the most crucial factors affecting patient compliance, treatment period, and efficacy. A total of ten studies reported the AEs in the present meta-analysis. The previous meta-analysis reported that local reactions resulted more from the drug solution, while non-standardized allergen extract caused systematic reactions [16]. In this study, we analyzed every injection reaction, and the results revealed that local swelling or erythema was more likely to occur in the ILIT group. Nevertheless, all these local events could be alleviated with or without medication. No statistical difference was observed in the rate of other AEs between the ILIT and placebo groups. These findings suggested that the intralymphatic delivery of allergen extract was safe and feasible.

The limitation of this systematic review and meta-analysis is the substantial heterogeneity and risk of bias in included studies. The data in included studies are too limited to perform a comprehensive subgroup analysis. More trials with long-term follow-up and large sample sizes are still in demand.

## 5. Conclusions

In conclusion, this systematic review and meta-analysis suggested that ILIT might be an alternative immunotherapy strategy for AR patients. Evidence from the current researches validated the safety and effectiveness of ILIT. ILIT was advantageous in improving the clinical symptoms of AR and reducing the need for medications. Moreover, the preseasonal booster injection had a positive impact on CSMS improvement. Future clinical trials involving perennial AR and trials with long-term follow-up and large sample sizes were recommended.

## Figures and Tables

**Table 1:** Study characteristics and participants.

First author, published year	Country	Trial ID	Centers	Enrolled period	Allergen	No. of participants		No. of females		Total dosage	Booster dose	Injection interval (weeks)	FU
						Active group	Placebo group	Active group	Placebo group				
Hellkvist, 2018 [29]	Sweden	2009-016815-39	2	2012 to 2015	Birch and grass pollen	24/30	27/30	6	10	3000 SQU	0	4	36-63w
Hellkvist, 2022 [31]	Sweden	NCT02679105/NCT02975479	3	2015 and 2016	Grass pollen	29/29	29/31	9	10	7000/9000/14000 SQU	0	4-7	8mo
Hjalmarsson, 2022 [30]	Sweden	NCT04296474	2	2012 to 2014	Birch and grass pollen	20/26	14/28	6	5	3000 SQU	0	4	5-7y
Hylander, 2016 [32]	Sweden	2009-016815-39	1	September 2010 to September 2011	Birch and/or grass pollen	20/21	15/15	8	6	3000 SQU	0	3-4	36w
Konradsen, 2020 [13]	UK	NCT03394508	1	January 1, 2013, and December 31, 2015	Birch and/or grass pollen	27/31	12/13	8	2	3000/4000 SQU	1	4-5	92-104w
Park, 2021 [15]	Korea	NCT02665754	1	2016 to December 2018	D. farinae, D. pteronyssinus, dogs, and cats	17/19	13/13	11	8	NA	0	4	48-50w
Patterson, 2016 [33]	USA	NCT01982474	1	December 2013 to February 2014	Grass pollen	7/7	8/8	Na	Na	400 PNU/mL	0	4	8w
Senti, 2012 [38]	Switzerland	NCT00718679	1	August to September 2008	Cat dander	8/12	5/8	8	6	14 *μ*g	0	4	43w
Skaarup, 2021 [34]	Denmark	2012-005227-33	1	2013	Grass pollen	23/24	12/12	12	5	3000/4000 SQU	1	4	144w
Terada, 2020 [35]	Japan	NA	2	NA	Japanese cedar pollen	12/12	6/6	8	4	60 JAU	0	4	130w
Thompson, 2020 [36]	USA	NA	1	Mountain cedar allergy season of 2018 to 2019	Mountain cedar pollen	11/11	10/10	7	5	3 : 2000 w/v	0	4	15-19w
Weinfeld, 2020 [39]	Sweden	NCT04210193	1	Autumn of 2014	Birch and/or grass pollen	13/13	Na	Na	Na	3000/4000 SQU	1	4	84w
Witten, 2013 [37]	Denmark	NA	1	Na	Grass pollen	25/30	13/15	6	5	3000/6000 SQU	0	2	35w

ILIT: intralymphatic immunotherapy; FU: follow-up time; SQU: standardized quality units; PNU: protein nitrogen units; JAU: Japanese allergy units; NA: not available.

## Data Availability

All data relevant to the study are included in the article or uploaded as supplementary information. No more additional data is available.

## Supplementary Materials

Supplementary Materials Supplementary Figure S1: subgroup analysis of CSMS by different dosages. Figure S2: subgroup analysis of CSMS by booster injection excluded Skaarup, S. H. 2021. Figure S3: subgroup analysis of CSMS by injection interval excluded Skaarup, S. H. 2021. Figure S4: the funnel plot of studies included in the meta-analysis of CSMS. Figure S5: subgroup analysis of VAS by different dosages. Figure S6: subgroup analysis of VAS by booster injection. Figure S7: the funnel plot of studies included in the meta- analysis of VAS. Figure S8: the funnel plot of studies included in the meta- analysis of RQLQ. Figure S9: the funnel plot of studies included in the meta- analysis of SPT.
