# Supplementary material for: Evaluation of Intralymphatic Immunotherapy in Allergic Rhinitis Patients: A Systematic Review and Meta-analysis
Source: Mediators Inflamm. 2023 May 8;2023:9377518. doi: 10.1155/2023/9377518 (PMC10185423; doi:10.1155/2023/9377518)
Supplement: Supplementary Materials — Supplementary Figure S1: subgroup analysis of CSMS by different dosages. Figure S2: subgroup analysis of CSMS by booster injection excluded Skaarup, S. H. 2021. Figure S3: subgroup analysis of CSMS by injection interval excluded Skaarup, S. H. 2021. Figure S4: the funnel plot of studies included in the meta-analysis of CSMS. Figure S5: subgroup analysis of VAS by different dosages. Figure S6: subgroup analysis of VAS by booster injection. Figure S7: the funnel plot of studies included in the meta- analysis of VAS. Figure S8: the funnel plot of studies included in the meta- analysis of RQLQ. Figure S9: the funnel plot of studies included in the meta- analysis of SPT. [file 9377518.f1.docx]

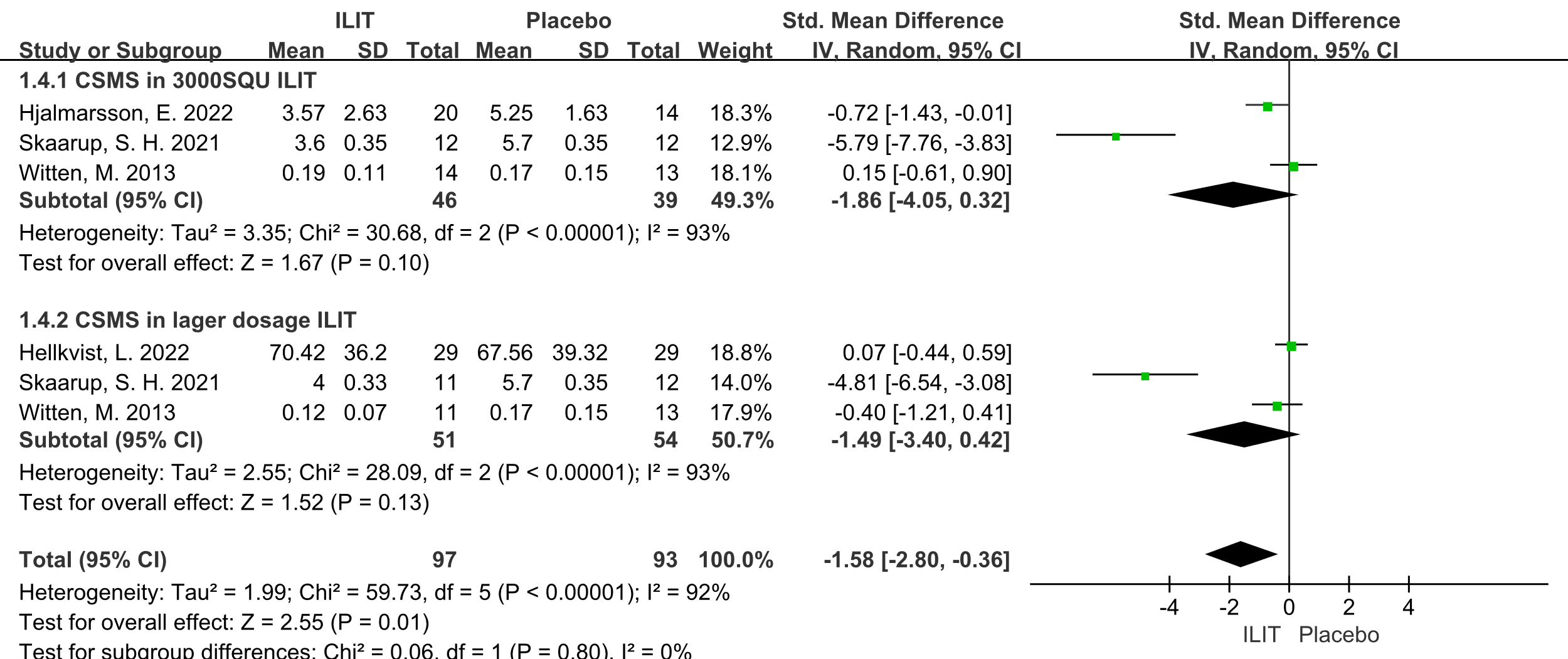
Figure S1 Subgroup analysis of CSMS by different dosages.


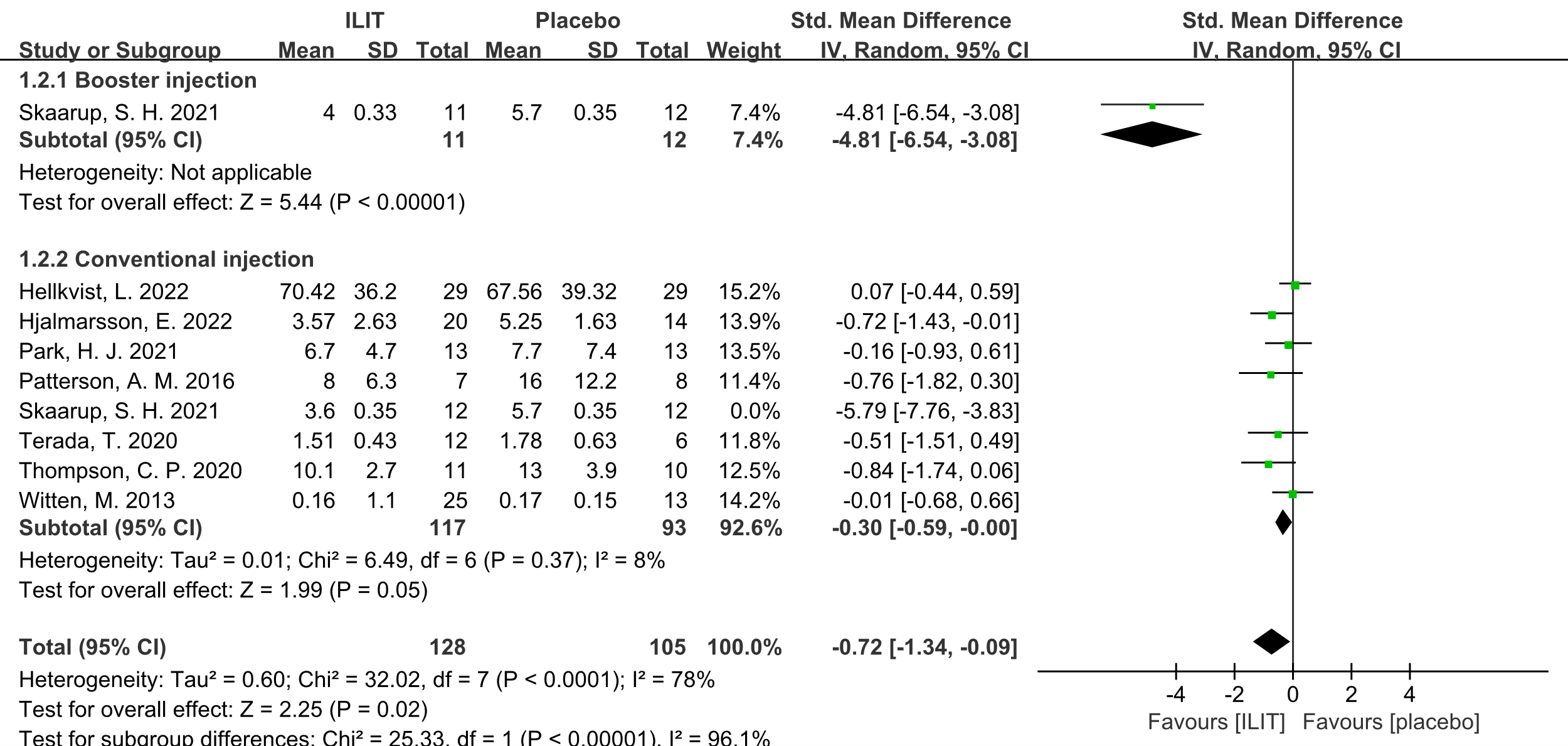


Figure S2 Subgroup analysis of CSMS by booster injection excluded Skaarup, S. H. 2021.


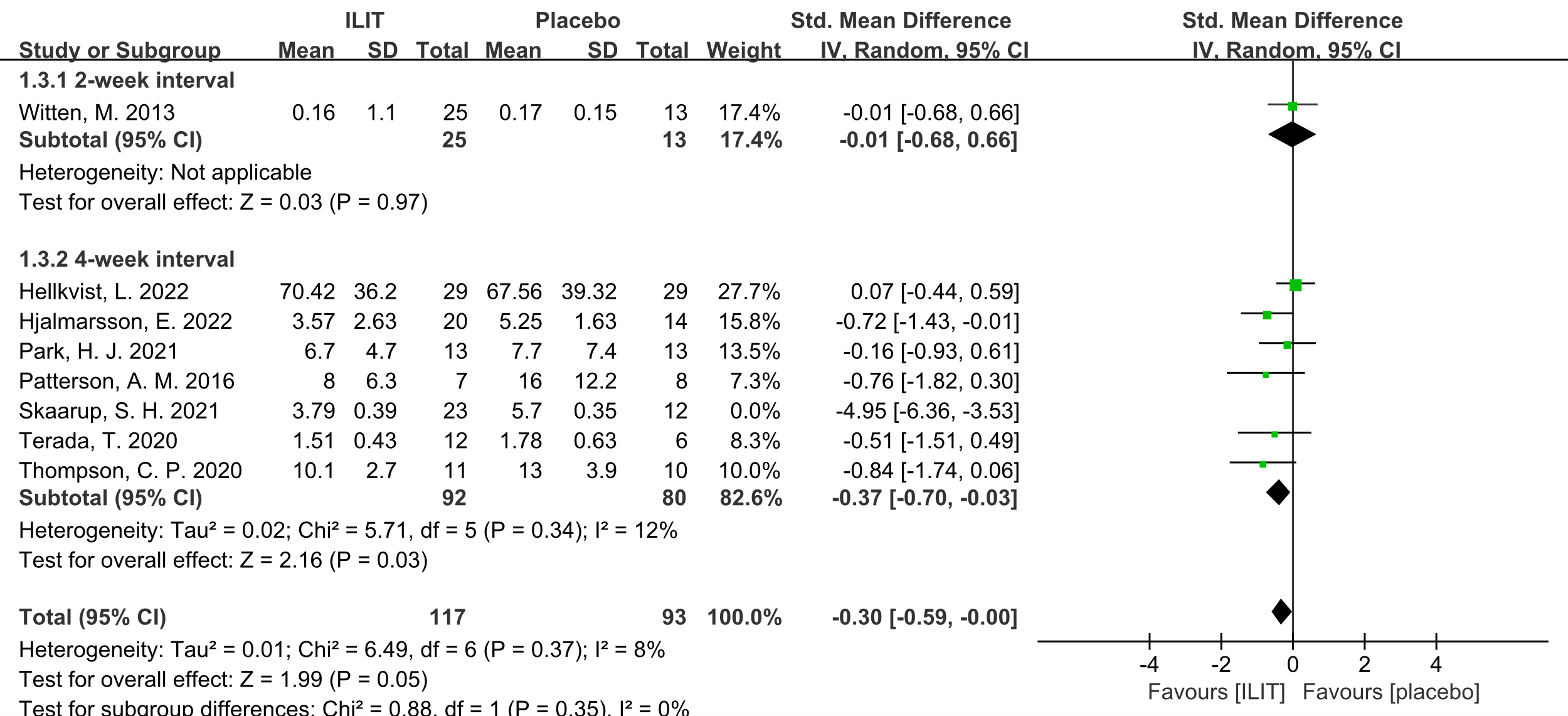


Figure S3 Subgroup analysis of CSMS by injection interval excluded Skaarup, S. H. 2021.


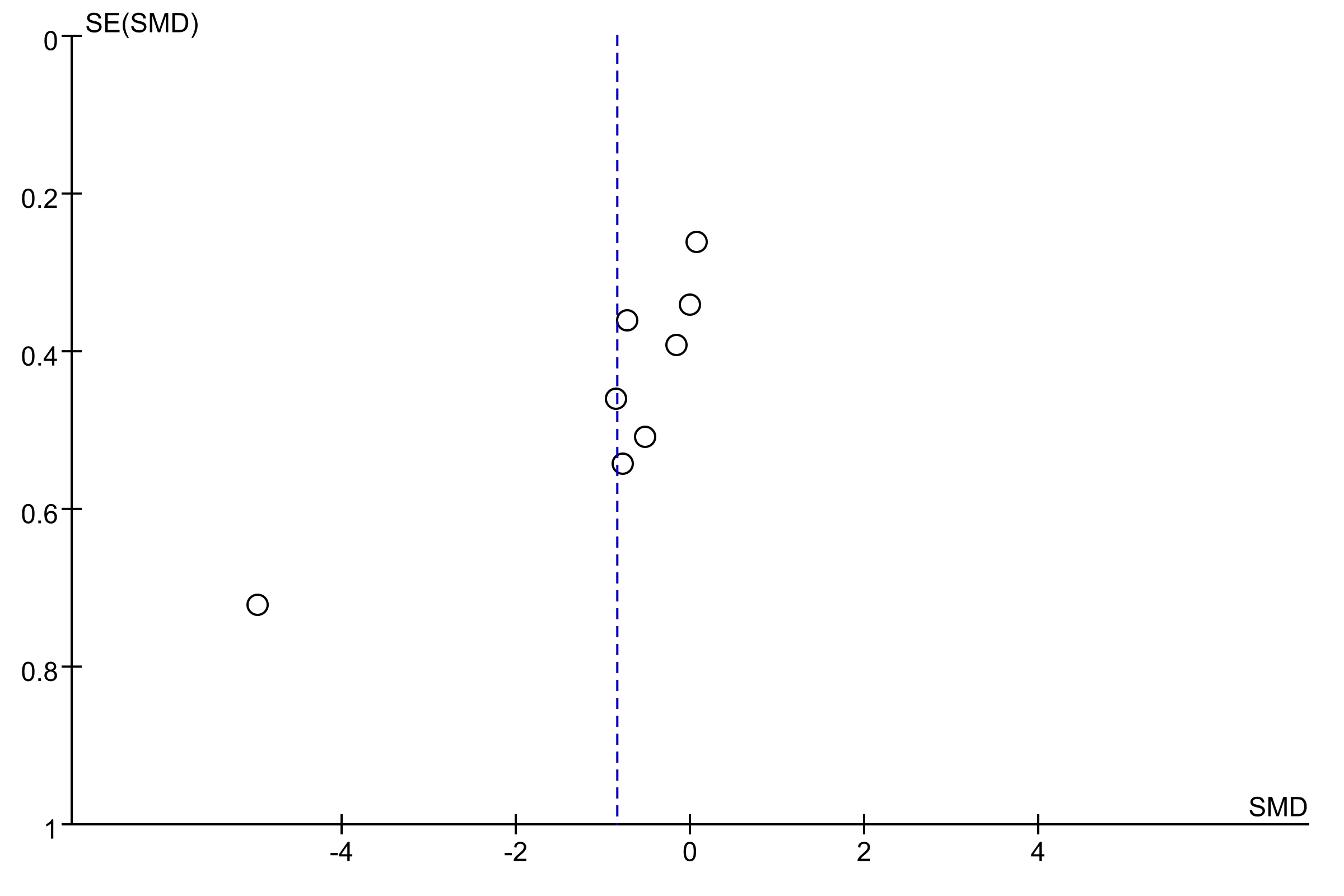
Figure S4 The funnel plot of studies included in the meta- analysis of CSMS

Figure
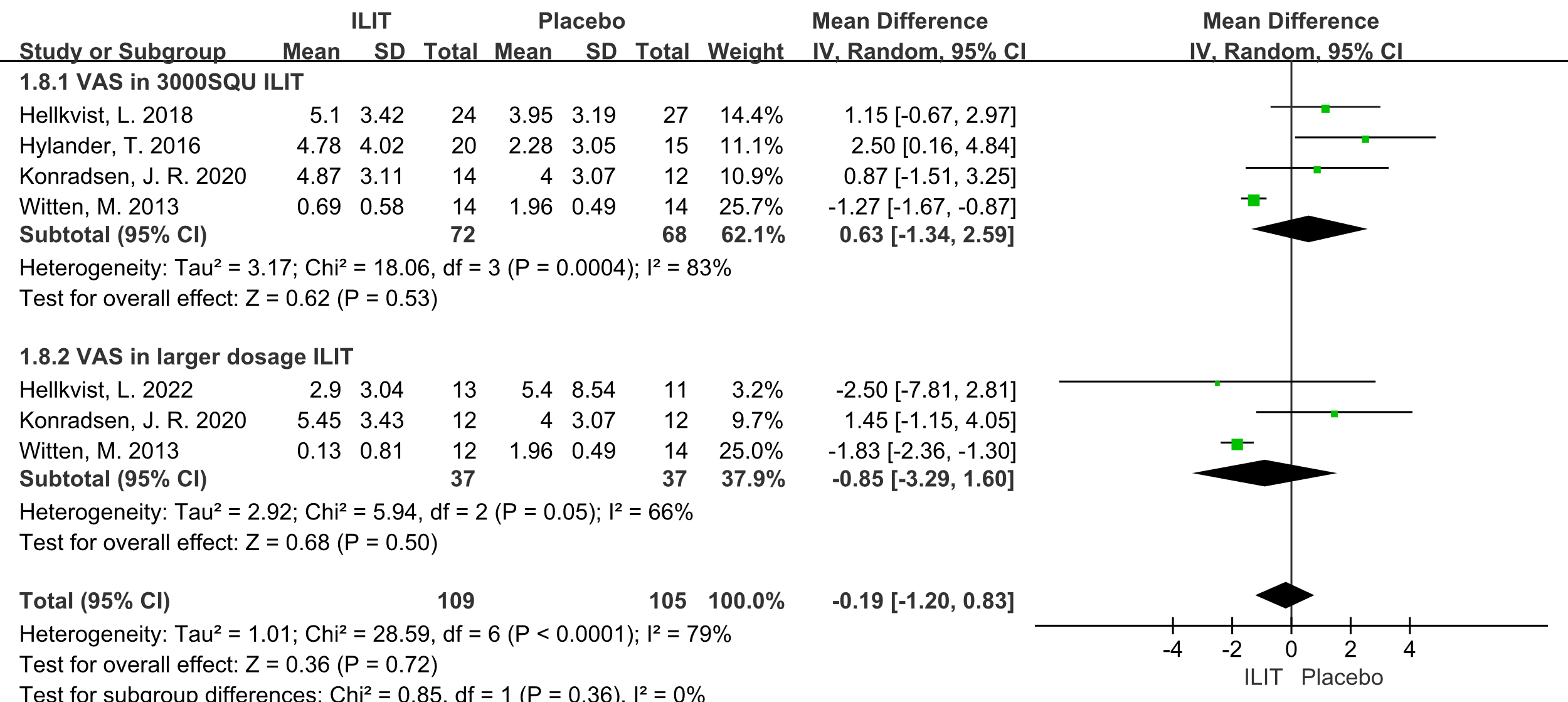
S5 Subgroup analysis of VAS by different dosages.


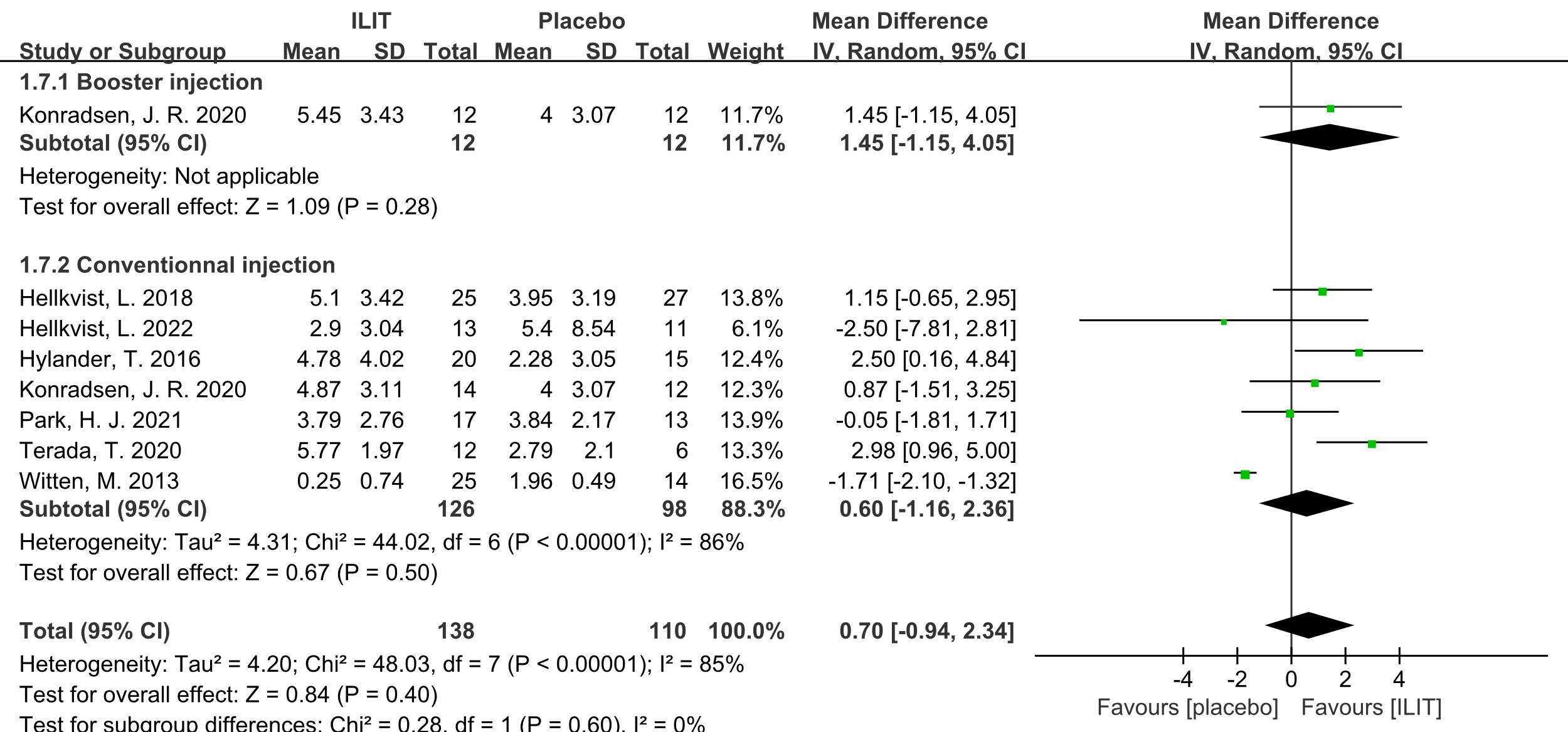


Figure S6 Subgroup analysis of VAS by booster injection.

Figure
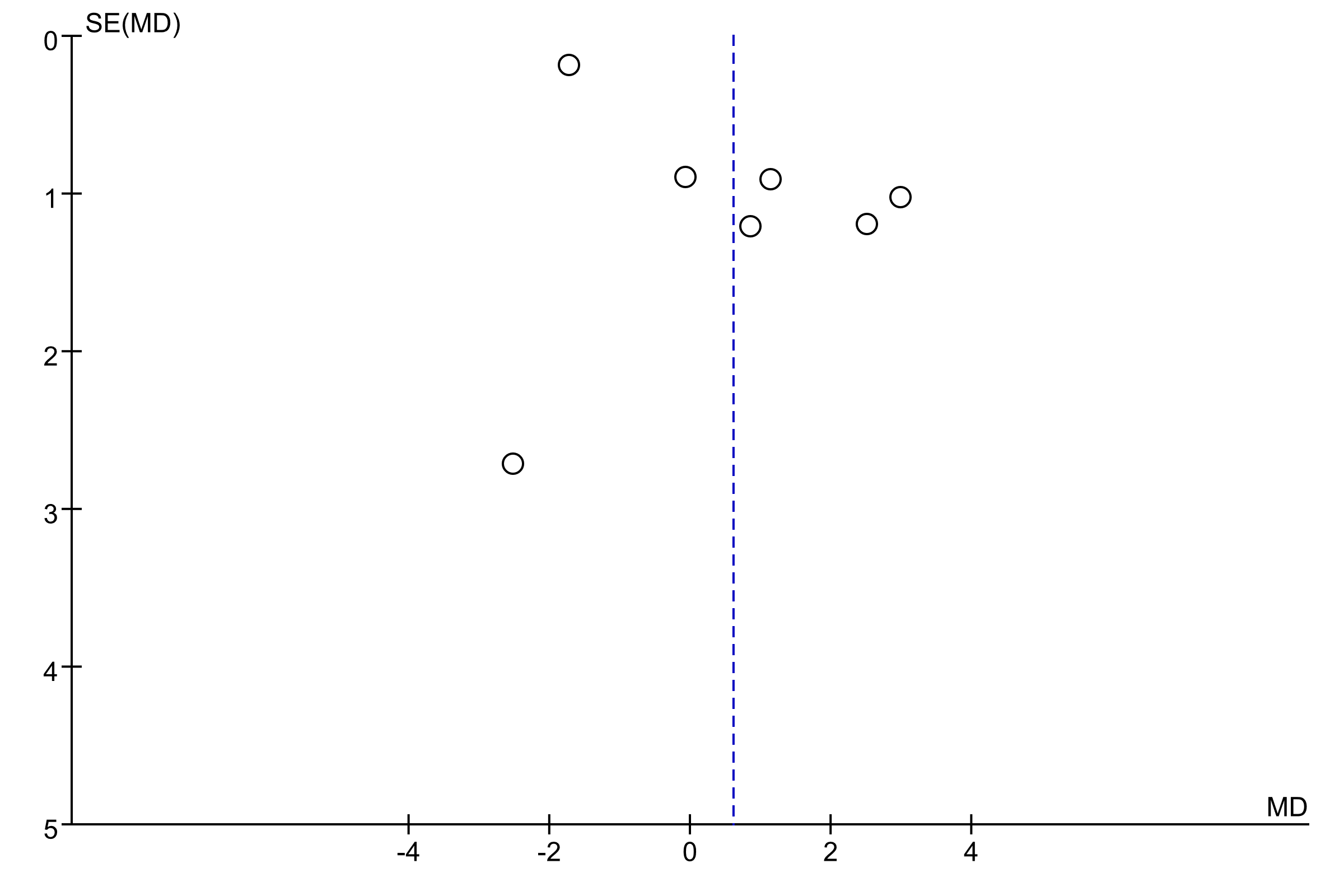
S7 The funnel plot of studies included in the meta- analysis of VAS


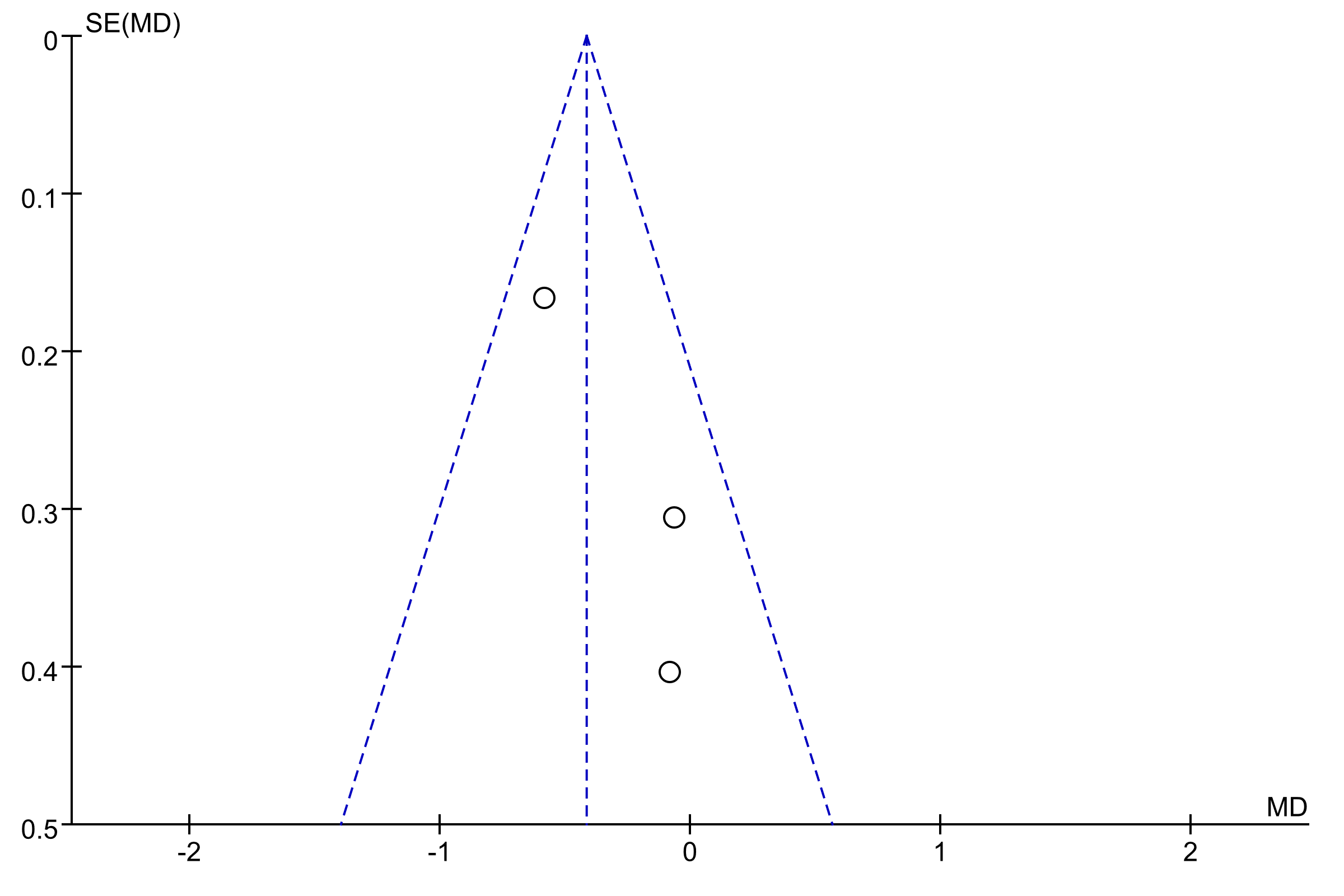


Figure S8 The funnel plot of studies included in the meta- analysis of RQLQ


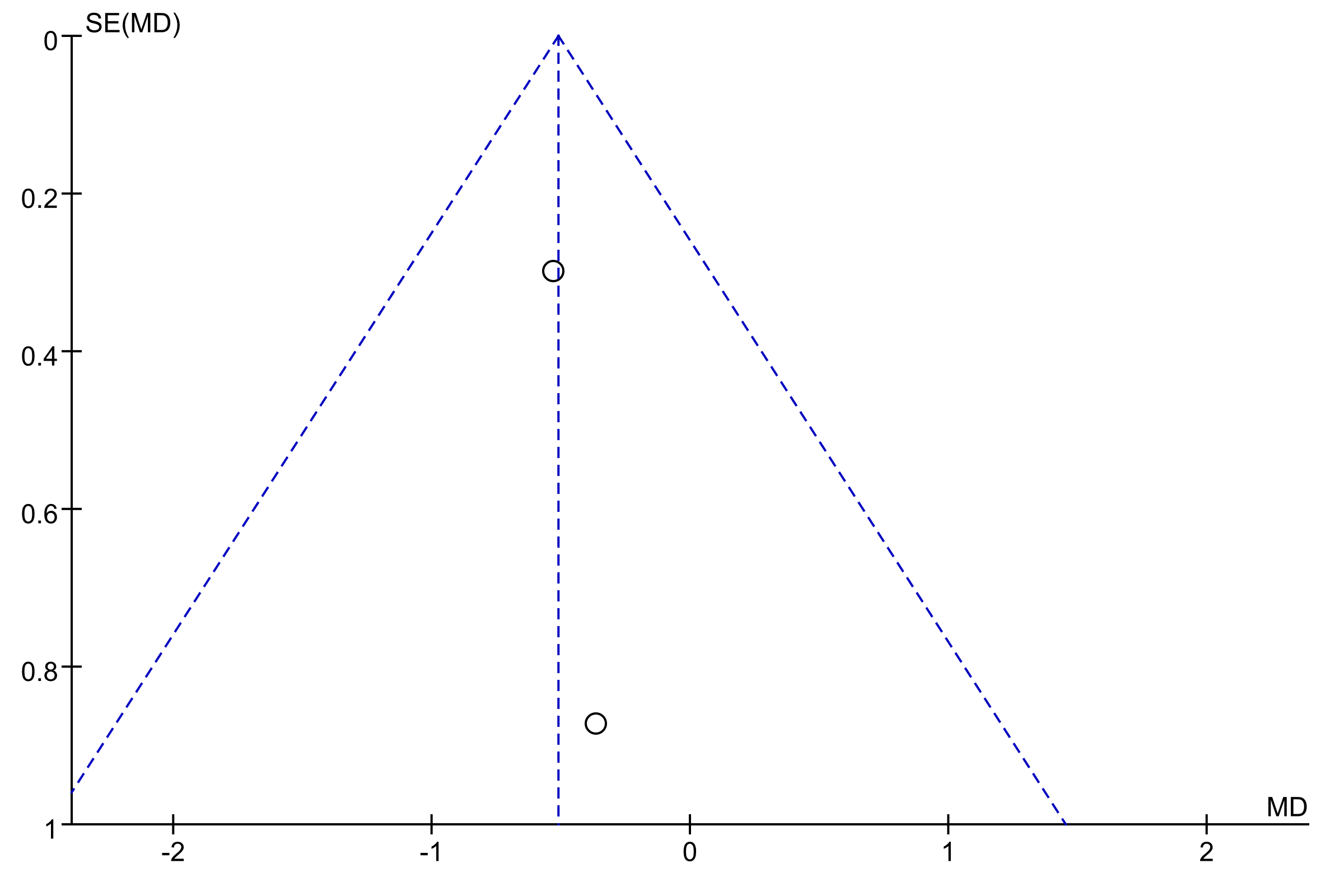


Figure S9 The funnel plot of studies included in the meta- analysis of SPT
